# Validity of classification of distal radial fractures in the Swedish fracture register

**DOI:** 10.1186/s12891-021-04473-5

**Published:** 2021-06-26

**Authors:** Malena Bergvall, Carl Bergdahl, Carl Ekholm, David Wennergren

**Affiliations:** 1 Department of Orthopaedics, Sahlgrenska University Hospital, SE-413 45 Göteborg, Gothenburg/Mölndal, Sweden; 2 Institute of Clinical Sciences, Sahlgrenska Academy, University of Gothenburg, Gothenburg, Sweden

**Keywords:** Distal radial fracture, Classification, AO/OTA classification, Fracture register, Validity, Accuracy, Agreement

## Abstract

**Background:**

Distal radial fractures (DRF) are one of the most common fractures with a small peak in incidence among young males and an increasing incidence with age among women. The reliable classification of fractures is important, as classification provides a framework for communicating effectively on clinical cases. Fracture classification is also a prerequisite for data collection in national quality registers and for clinical research. Since its inception in 2011, the Swedish Fracture Register (SFR) has collected data on more than 490,000 fractures. The attending physician classifies the fracture according to the AO/OTA classification upon registration in the SFR. Previous studies regarding the classification of distal radial fractures (DRF) have shown difficulties in inter- and intra-observer agreement. This study aims to assess the accuracy of the registration of DRF in adults in the SFR as it is carried out in clinical practice.

**Methods:**

A reference group of three experienced orthopaedic trauma surgeons classified 128 DRFs, randomly retrieved from the SFR, at two classification sessions 6 weeks apart. The classification the reference group agreed on was regarded as the gold standard classification for each fracture. The accuracy of the classification in the SFR was defined as the agreement between the gold standard classification and the classification in the SFR. Inter- and intra-observer agreement was evaluated and the degree of agreement was calculated as Cohen’s kappa.

**Results:**

The accuracy of the classification of DRF in the SFR was kappa = 0.41 (0.31–0.51) for the AO/OTA subgroup/group and kappa = 0.48 (0.36–0.61) for the AO/OTA type. This corresponds to moderate agreement. Inter-observer agreement ranged from kappa 0.22–0.48 for the AO/OTA subgroup/group and kappa 0.48–0.76 for the AO/OTA type. Intra-observer agreement ranged from kappa 0.52–0.70 for the AO/OTA subgroup/group and kappa 0.71–0.76 for the AO/OTA type.

**Conclusions:**

The study shows moderate accuracy in the classification of DRF in the SFR. Although the degree of accuracy for DRF appears to be lower than for other fracture locations, the accuracy shown in the current study is similar to that in previous studies of DRF.

## Background

Distal radial fractures (DRF) are one of the most common fractures with a small peak in incidence among young males and an increasing incidence with age among women [1–5]. The reliable classification of fractures is important, as classification provides a framework for communicating effectively on clinical cases. Fracture classification is also a prerequisite for data collection in national quality registers and for clinical research. There are numerous classification systems for the classification of DRF, of which Frykman, Fernandez, Universal, Melone, Mayo and AO/OTA (Arbeitsgemeinschaft für Osteosynthesefragen/Orthopaedic Trauma Association) are the most commonly used [6–8]. Each of these classification systems has a different focus, but none has shown superiority over the others regarding classification reliability [8–11].

The Swedish Fracture Register (SFR) is a unique national quality register where all orthopaedic fractures, regardless of treatment, are registered [12]. Since the start in 2011, more than 490,000 fractures have been registered with their fracture classification, subsequent treatment(s) and outcome [13]. Data collection is web based and filed by the attending orthopaedic surgeon. As a result the majority of fractures in the SFR are primarily registered by junior residents at the A&E, with limited time and no specific training in fracture classification [12]. In the SFR, the classification of fractures is made according to the AO/OTA (2007 version), since it is comprehensive and designed to have a similar structure for all the bones in the body [14, 15]. Four studies have examined the accuracy of fracture classification in the SFR (tibia, humerus, ankle and femur fractures) [16–19]. The results of these studies are in accordance with previous studies and showed kappa values corresponding to moderate to substantial agreement according to Landis and Koch [20]. However, the classification of DRF appears to be particularly difficult [21]. Previous studies regarding the classification of DRF in the AO/OTA system have shown inter-observer agreement lower than that for other end-segmental fractures, especially at group and subgroup level [8, 9, 11, 21–24]. The accuracy of the classification of DRF in the SFR has not previously been evaluated, nor has the classification of DRF according to the AO/OTA system been evaluated when carried out in daily practice outside a test setting.

**Fig. 1:**
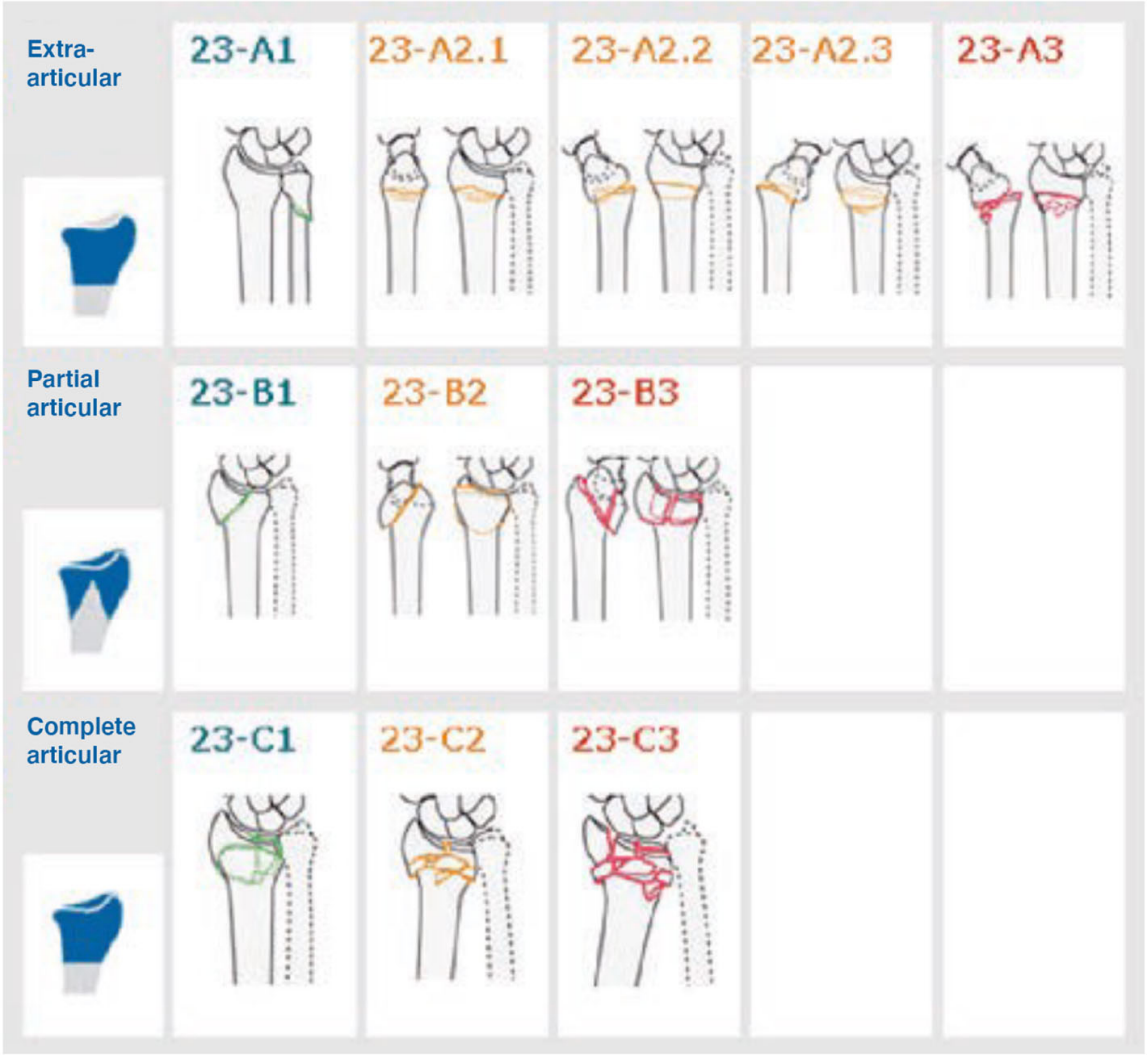
The AO/OTA classification of distal radial fractures as shown in the Swedish Fracture Register

In the AO/OTA system, fracture classes are defined by a number of Bolean questions (Yes or No) and certain fractures can be separated by only one such defining question, for example, whether or not there is an intra-articular fracture line (Fig. 1). In a previous study of the validity of the classification of humeral fractures, fracture classes were called *related* fracture classes if there was only one feature separating two fracture classes. If this feature is not obvious on the radiographs, observers may disagree on the classification. Fracture classes differing by two or more defining questions are regarded as *unrelated.* This method of defining related classification groups can be used to interpret low inter-observer agreement [17].

The primary aim of this study was to assess the accuracy of the classification of distal radial fractures in adults as carried out in clinical practice in the SFR and to examine the inter- and intra-observer reliability of the AO/OTA classification regarding DRFs. The secondary aim was to examine whether the majority of disagreements were between *related* fracture groups.

## Methods

**Fig. 2:**
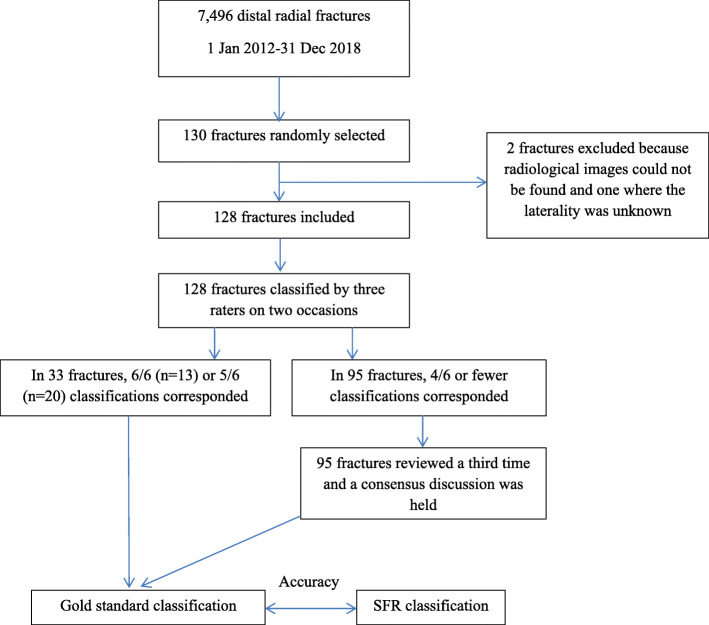
Flow chart showing how the study was conducted

Following a sample size calculation, 130 patients with a DRF were randomly allocated among the 7496 DRFs registered in the SFR at Sahlgrenska University Hospital during the time period 2012–2018 (Fig. 2). Inclusion criteria were patients aged 16 years and above, registered with a distal radial fracture in the SFR and treated at the Sahlgrenska University Hospital during the time period 2012–2018. Exclusion criteria were patients younger than 16 years, if no radiological images could be found and if the laterality was unknown. Two patients were excluded, as radiological images could not be found for one patient and the laterality was unknown in another patient with bilateral wrist fractures. The medical charts were reviewed for the remaining 128 patients and the radiological images available at the time of registration in the SFR were extracted and anonymised by one of the authors (MB).

The radiological images for each fracture were presented to a reference group of three orthopaedic surgeons (CB, CE, DW). In clinical practice, at the department where the study was conducted, CT scan is not performed in all cases but in selected cases when the attending orthopaedic surgeon decides that CT scan is necessary for decision on the treatment of the fracture. Therefore, in the study, the same radiographic modalities that had been present when the fracture was classified upon registration in the Swedish Fracture Register, was used in the study and presented to the reference group. For 108 patients, only plain radiographs, comprising anteroposterior (AP) and lateral views, were available. Plain radiographs with an additional CT scan were available in 16 patients and only a CT scan was available for four patients. Both pre- and post-reduction images were included if available. Each fracture was classified by the members of the reference group at two different seminars 6 weeks apart, meaning that every fracture was classified six times. The observers sat apart and were unable to interact with each other. If 6/6 or 5/6 classifications for a specific fracture corresponded, that classification was regarded as the gold standard classification for that fracture. A third seminar was held to openly discuss the fractures with four or fewer corresponding classifications. In this way, the gold standard classification was defined for all 128 fractures (Fig. 2). The classification registered in the SFR was then compared with the gold standard classification and accuracy, defined as agreement between the classification in the SFR and the gold standard classification, was calculated.

In the SFR, fractures are classified according to the AO/OTA classification system (11). For DRFs, the group level is used (A1-C3), except for A2 fractures which are expanded to subgroups (A2.1-A2.3), as these fractures are common and the subgroup classification allows discrimination between fractures with a dorsal angulation (Colles/A2.2) and a volar angulation (Smith/A2.3). This makes a total of 11 eligible fracture classes in the SFR and these 11 fracture classes were therefore used in the study (Fig. 1).

To examine the concept of related fracture classes, the classification made in the SFR and the gold standard classification for each fracture were plotted in a cross-table. This visualises the fracture groups between which disagreements occurred.

### Statistic

Percentage agreement and Cohen’s kappa were calculated for inter- and intra-observer agreement, as well as accuracy, defined as agreement between the classification in the SFR and the gold standard classification. The kappa values were interpreted according to Landis and Koch; 0–0.20 slight agreement, 0.20–0.40 fair agreement, 0.40–0.60 moderate agreement, 0.60–0.80 substantial agreement and 0.80–1 excellent or almost perfect [25, 26].

Based on previous studies, a kappa value of approximately 0.5 was expected [16–19]. To achieve a 95% confidence interval that did not span more than one category on the scale defined by Landis and Koch, a relative error of 20% (i.e. kappa+/− 0.1) was accepted [20]. Based on these assumptions, a sample size calculation which rendered 130 patients was made. SAS software v 9.4 SAS Institute Inc., Cary, NC, USA was used for statistical analysis.

### Ethics

The study was performed according to the Helsinki declaration, approved by the Swedish Ethical Review Authority, reference number 2019–04312, and performed in accordance with this approval.

## Results

There were 128 patients in the study, of which 33 were men and 95 were women. The median age was 61 years (range 18–97) for all patients, 56 years (range 18–79) for men and 62 years (range 24–97) for women (Table 1).

**Table 1:** Distribution of patients with distal radial fractures according to age, gender and type of fracture. The reference group classified three cases as no fracture and one case as pathological; they are not included in this table

	Women	Men	Total
Median age (range)	62.5 (24–97)	56 (18–79)	60 (18–97)
all fractures	*n* = 92	*n* = 32	*n* = 124
Median age (range)	62.5 (24–95)	67.5 (23–79)	64.5 (23–95)
distal radial fractures (23A)	*n* = 52	*n* = 10	*n* = 62
Median age (range)	59 (36–66)	38.5 (18–72)	48 (18–72)
distal radial fractures (23B)	*n* = 3	*n* = 4	*n* = 7
Median age (range)	63 (27–97)	63 (18–71)	58 (18–97)
distal radial fractures (23C)	*n* = 37	*n* = 18	*n* = 55

In 33 fractures, 6/6 or 5/6 classifications by the reference group corresponded after the two classification seminars and this classification was regarded as the gold standard classification. In the remaining 95 fractures, four or fewer classifications corresponded and gold standard classification was achieved through consensus discussion for these fractures (Table 2).

**Table 2:** Distribution of classification agreement of the three raters of the reference group regarding distal radial fractures

Number of corresponding classifications	Number of fractures
6/6	13
5/6	20
4/6	43
3/6	39
2/6	13

The gold standard classification included 62 A fractures, seven B fractures and 55 C fractures (Table 3).

**Table 3:** Number of fractures for each fracture class according to the gold standard classification regarding distal radial fractures. The consensus group classified three cases as no fracture and one fracture as a pathological fracture, making the total number of fractures in Table 3 124. A2 fractures include 22 A2.1, 29 A2.2 and 3 A2.3

	1	2	3	Total
23A	0	54	8	62
23B	5	0	2	7
23C	27	7	21	55

The percentage agreement between gold standard and the SFR was 50% for the AO/OTA group (4 signs), including the A2 subgroups (5 signs), and 70% for the AO/OTA type (3 signs) (Table 4). Cohen’s kappa coefficient for accuracy, defined as agreement between the gold standard classification and the classification in the SFR, was 0.41 (95% CI: 0.31–0.51) for the AO/OTA group (including the A2 subgroups) and 0.48 (95% CI: 0.36–0.61) for the AO/OTA type. This corresponds to moderate agreement according to Landis and Koch [20].

**Table 4:** Accuracy, defined as the agreement between the classification in the SFR compared with the gold standard classification regarding distal radial fractures. As presented in Fig. 1, the AO/OTA group includes the 23A2 subgroups

	Accuracy	
	PA	Kappa (95% CI)
AO/OTA group, including A2 subgroup (4–5 signs)	50%	0.41 (0.31–0.51)
AO/OTA type (3 signs)	70%	0.48 (0.36–0.61)

*PA* Percentage of agreement, *GS* Gold standard classification

The inter-observer agreement between the three raters at the two seminars ranged from kappa 0.22–0.48 (fair to moderate) for the AO/OTA group (including the A2 subgroups) and from kappa 0.48–0.76 (moderate to substantial) for the AO/OTA type (3 signs) (Table 5). The intra-observer agreement ranged from kappa 0.52–0.70 (moderate to substantial) for the AO/OTA group (including the A2 subgroups) and kappa 0.71–0.76 (substantial) for the AO/OTA type (Table 6).

**Table 5:** Inter-observer agreement regarding distal radial fractures comparing the three raters at the two classification seminars. As presented in Fig. 1, the AO/OTA group includes the 23A2 subgroups

	Inter-observer agreement					
	Rater 1 vs Rater 2		Rater 1 vs Rater 3		Rater 2 vs Rater 3	
	Seminar 1 Kappa (95% CI)	Seminar 2 Kappa (95% CI)	Seminar 1 Kappa (95% CI)	Seminar 2 Kappa (95% CI)	Seminar 1 Kappa (95% CI)	Seminar 2 Kappa (95% CI)
AO/OTA group, including A2 subgroup (4–5 signs)	0.34 (0.24–0.44)	0.48 (0.38–0.58)	0.22 (0.14–0.31)	0.29 (0.20–0.37)	0.28 (0.20–0.37)	0.35 (0.26–0.44)
AO/OTA type (3 signs)	0.48 (0.36–0.60)	0.76 (0.66–0.87)	0.51 (0.39–0.63)	0.69 (0.58–0.80)	0.62 (0.51–0.73)	0.71 (0.61–0.82)

**Table 6:** Intra-observer agreement regarding distal radial fractures comparing the classification of each rater at the two different classification seminars. As presented in Fig. 1, the AO/OTA group includes the 23A2 subgroups

	Intra-observer agreement					
	Rater 1		Rater 2		Rater 3	
	PA	Kappa (95%CI)	PA	Kappa (95%CI)	PA	Kappa (95%CI)
AO/OTA group including A2 subgroup (4–5 signs)	60%	0.52 (0.42–0.62)	63%	0.54 (0.45–0.64)	75%	0.70 (0.62–0.79)
AO/OTA type (3 signs)	87%	0.75 (0.64–0.86)	83%	0.71 (0.60–0.81)	84%	0.76 (0.66–0.85)

*PA* Percentage of agreement, *CI* Confidence interval

Table 7 visualises the fracture groups between which disagreements between the classification in the SFR and the gold standard classification occurred. In the cross-table, the diagonal represents cases with full agreement, whereas numbers outside the diagonal represent disagreements. When the difference between two fracture groups depended on the answer to only one defining question, the fracture groups were regarded as *related* (green boxes in Table 7), otherwise *unrelated* (red boxes in Table 7). When gold standard classification was compared with the initial classification in the SFR, full agreement was seen in 64 of 128 fractures. Of the remaining 64 fractures, there was disagreement within *related* fracture classes in 38 (59%) of the fractures and between *unrelated* fracture classes in 26 (41%) fractures (Table 7).

**Table 7:**
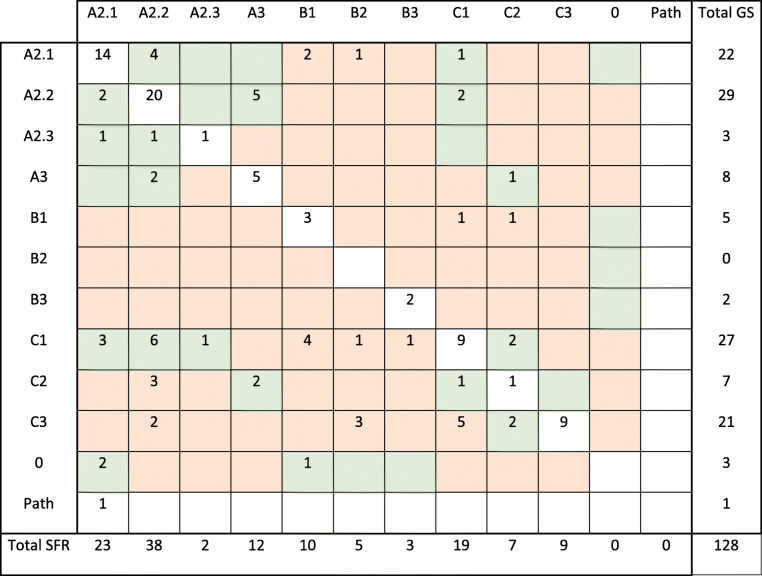
Cross-tab that shows how the distal radial fractures in the study were classified in the SFR (columns) and the gold standard classification (rows) respectively. The boxes on the diagonal (white boxes) represent cases with full agreement between the classification in the SFR and the gold standard classification, whereas boxes outside the diagonal represent disagreements. When the difference between two fracture groups depends on the answer to only one defining question, the fracture groups are regarded as *related* and the boxes are green. When two fracture groups are separated by the answer to more than one defining question, the fracture groups are regarded as *unrelated* and the boxes are red

Path = pathological fracture, 0 = the reference group classified the case as no fracture, SFR = the classification in the Swedish Fracture Register, GS = gold standard classification

## Discussion

The primary aim of this study was to examine the accuracy of the AO/OTA classification of distal radial fractures as it is used in the SFR. The accuracy, defined as agreement between the classification in the SFR and the gold standard classification, was moderate, regarding both the AO/OTA group (including the A2 subgroups) (kappa 0.41) and the type (kappa 0.48).

The purpose of the study was to assess the accuracy of the classification of distal radial fractures in adults as carried out in clinical practice in the SFR and to examine whether the majority of disagreements were between *related* fracture groups. The present study differs from previous studies of the validity of classification of DRFs since it compares a consensus classification (the gold standard classification) with the classification made in everyday clinical practice by clinicians with varying experience (the classification in the SFR). Despite this, the agreement between the classification in the SFR and the gold standard classification is in accordance with the inter-observer kappa values in previous studies. Weaver et al. reported kappa values of 0.45 and 0.24 (for types and groups respectively), Yinjie et al. of 0.47 for groups and Plant et al. of 0.56 and 0.29 (for types and groups respectively), which are all in the same range as in the present study [9, 11, 24]. In the present study, the inter-observer reliability was moderate (kappa 0.48) to substantial (kappa 0.76) for the AO/OTA type, but it dropped to moderate (kappa 0.48) to fair (kappa 0.22) at the group/subgroup level. The classification of DRFs has been shown to be more difficult than other end-segmental fractures in the AO/OTA classification [21]. The low inter- and intra-observer agreement regarding the classification of DRFs is not exclusive to the AO/OTA system. Moreover, the classification systems of Frykman, Olders, Fernandez and Melone have shown low inter- and intra-observer agreement [8, 9, 11].

A CT scan examination is currently widely used to assess the fracture details and to facilitate fracture classification. A number of studies have evaluated the value of this kind of investigation of the AO/OTA classification reliability. Flinkkilä et al., Kleinlugtenbelt et al. and Arealis et al. found that, although a CT scan improved the ability to detect intra-articular fracture lines, it added little value to improving inter-observer reliability [6, 10, 27]. As a seemingly natural consequence of this, both Flinkkilä et al. and Kleinlugtenbelt et al. found that the proportion of fractures classified as intra-articular was higher when a CT scan was used [7, 27]. The explanation for the lack of clear effect of a CT scan on the reliability in these studies could be the so-called coastline paradox, which states that the length of a coastline varies with the measurement scale or that complexity increases with a smaller measurement scale. In cases in which a CT scan is used to determine fracture classification, some questions are resolved (e.g. whether the fracture is intra-articular). However, due to more details becoming visible, new questions may arise that could confound the classification.

It is apparent that, for the 64 fractures in the present study where the classification of DRFs in the SFR and the gold standard classification diverged, the majority of the divergences were between *related* fracture classes. These fracture classes are separated by only one defining question. This question could relate to the presence of a fracture line, which may be difficult to determine, or the degree of comminution, which lacks a clear definition. The inclusion of subgroups (A2.1-A2.3) did not significantly lower the kappa value, which may be related to the defining questions for these subgroups being relatively clear-cut (volar, dorsal or no displacement). In the present study, when the classification was simplified from AO/OTA group/subgroup (4/5 signs) to AO/OTA type (3 signs), the agreement was not substantially improved (kappa 0.41 and 0.48 respectively). An explanation for this might be that, even though disagreements are often between *related* fracture classes, the *related* fracture classes are not always within the same fracture type. A previous study of the validity of humeral fracture classification showed that related fracture classes could be far apart on the pictorial chart of the classification scheme [17]. This is exemplified in the AO/OTA classification of DRF, where the one feature separating a C2 fracture from an A3 fracture is an intra-articular fracture line. In spite of this, A3 and C2 are far apart in the pictorial chart. Furthermore, kappa calculation does not take account of the degree of disagreement. Weighted kappa on the other hand is not suitable, as fracture classification is a nominal scale. A disagreement in the classification of fractures close to the border of two related categories may not necessarily be of major significance or clinical relevance, but it affects the kappa value. However, with the concept of related fractures, the present study shows full agreement or disagreement within related fracture classes between the SFR and the gold standard in 80% (102/128). Full disagreement (disagreement between *unrelated* fracture classes) between the SFR and the gold standard was only seen in 20% (26/128) (Table 7). This model for interpreting the results may explain why the kappa values do not increase considerably when simplifying the AO/OTA classification from group/subgroup to type.

### Strengths and limitations

The study design is in accordance with the quality criteria of Audige et al. and is similar to other validity studies made in the SFR [16–19, 25, 26]. The study population of 128 fractures is extensive and, as the study period extends over six years, the study is not affected by seasonal variations. The long study period also means that the junior residents at the A&E have been replaced several times, reducing the possible bias of individual skills. The study had no specific exclusion criteria (except age above 16 years) – all fractures were eligible regardless of treatment. No fractures were classified as A1 or B2 in the gold standard classification, however, all the other fracture groups were represented in the study. One possible bias is that all the fractures came from the same hospital, Sahlgrenska University Hospital, where many of the co-workers understand the importance of correct registration. In future studies it would be of interest to study fractures treated at different departments affiliated with the SFR. This study reflects the classification made in real-life conditions, at the A&E by the attending orthopaedic surgeons, some with limited experience. The radiological images were not standardised or excluded in the event of poor quality. The fact that the quality of the images varied reflects clinical practice. There is no such a thing as a perfect classification system. Nor such a thing as “a perfect truth” in the interpretation of the system, more a “weighing of expert opinions”. Therefore, regarding classification of fractures, there will always be some disagreement between observers since it relies on both an interpretation of the radiological images as well as of the classification system used. It is possible to argue that the “gold standard” classification is arbitrary, but to our knowledge there is no better way to define the “correct” classification.

The question remains of why the classification of DRFs universally shows such low kappa values. It becomes apparent that, to improve the accuracy of wrist fracture classification, the classification systems need to be modified, based on defining questions that are well defined and easy to assess. Although simplifying the systems, e.g. reducing the AO/OTA system to types only (A, B, C), improves the agreement to some extent, this renders the classification meaningless for treatment selection, prognosis and scientific work. The current study shows kappa values that are similar to those in previous studies. However, the concept of related fracture classes, presented in the current study, offers some explanation to the background for the poor kappa values.

## Conclusion

The study shows moderate agreement between the AO/OTA classification used in clinical practice in the SFR and a gold standard classification. Although the degree of accuracy for the classification of DRFs appears to be lower than that for other fracture locations, the accuracy shown in the current study is similar to that in previous studies of DRF. Among the fractures where the classification in the SFR and the gold standard classification did not fully agree, the disagreements were between *related* fracture groups in 59%.

## Data Availability

The datasets generated during and analyzed during the current study are not publicly available due to that the datasets includes multiple extensive files with a large number of variables, but are available from the corresponding author on reasonable request.

## Abbreviations

AO: Arbeitsgemeinschaft für osteosynthesefragen
DRF: Distal Radial Fracture
OTA: Orthopaedic Trauma Association
SFR: Swedish Fracture Register
